# The ethical desirability of moral bioenhancement: a review of reasons

**DOI:** 10.1186/1472-6939-15-67

**Published:** 2014-09-16

**Authors:** Jona Specker, Farah Focquaert, Kasper Raus, Sigrid Sterckx, Maartje Schermer

**Affiliations:** 1 Department of Medical Ethics and Philosophy, ErasmusMC University Medical Center Rotterdam, P.O. Box 2040, 3000, CA, Rotterdam, The Netherlands; 2 Bioethics Institute Ghent, Department of Philosophy & Moral Sciences, Ghent University, Blandijnberg 2, 9000 Gent, Belgium

**Keywords:** Moral enhancement, ethical analysis, neuroethics

## Abstract

**Background:**

The debate on the ethical aspects of moral bioenhancement focuses on the desirability of using biomedical as opposed to traditional means to achieve moral betterment. The aim of this paper is to systematically review the ethical reasons presented in the literature for and against moral bioenhancement.

**Discussion:**

A review was performed and resulted in the inclusion of 85 articles. We classified the arguments used in those articles in the following six clusters: (1) why we (don’t) need moral bioenhancement, (2) it will (not) be possible to reach consensus on what moral bioenhancement should involve, (3) the feasibility of moral bioenhancement and the status of current scientific research, (4) means and processes of arriving at moral improvement matter ethically, (5) arguments related to the freedom, identity and autonomy of the individual, and (6) arguments related to social/group effects and dynamics. We discuss each argument separately, and assess the debate as a whole. First, there is little discussion on what distinguishes moral bioenhancement from treatment of pathological deficiencies in morality. Furthermore, remarkably little attention has been paid so far to the safety, risks and side-effects of moral enhancement, including the risk of identity changes. Finally, many authors overestimate the scientific as well as the practical feasibility of the interventions they discuss, rendering the debate too speculative.

**Summary:**

Based on our discussion of the arguments used in the debate on moral enhancement, and our assessment of this debate, we advocate a shift in focus. Instead of speculating about non-realistic hypothetical scenarios such as the genetic engineering of morality, or morally enhancing ‘the whole of humanity’, we call for a more focused debate on realistic options of biomedical treatment of moral pathologies and the concrete moral questions these treatments raise.

## Background

Should we develop and implement interventions that aim to improve people’s morality? Ever since the publication of two papers in a special issue of the *Journal of Applied Philosophy* in 2008 [1,2], the ethical desirability of moral bioenhancement has been the subject of intense debate. Whereas ‘traditional methods’ of moral betterment (such as upbringing, socialization and education) are arguably as old as humanity itself, the debate on moral bioenhancement focuses specifically on the desirability of the use of biomedical methods. Interventions that are being investigated in the literature range from various types of psychopharmaceuticals, deep brain stimulation (DBS), and genetic selection and engineering.

In a previous paper [3] we examine the different ways in which the concept of moral bioenhancement is used in the literature: what different authors understand its main goals to be, what would count as a success, and what kind of interventions would and would not fall within their proposed definitions. In this paper, we ask what reasons and arguments have so far been given in the debate on the ethical desirability of moral bioenhancement. We do this by mapping out the different arguments that have been presented in the debate up till now (see subsection ‘Arguments used in the debate’). We aim to provide a complete overview including both the main arguments in the debate as well as the less commonly voiced arguments. In the final critical appraisal section (see subsection ‘Critical appraisal of the current debate’), we analyze the kinds of arguments given, thereby critically assessing the issues and concerns that have been discussed, as well as identifying those issues and concerns that up to now have been neglected. This section represents our own interpretation and views concerning the debate and the arguments that are given. We argue for a shift in focus of the debate towards a discussion of more realistic interventions for specific target groups.

## Discussion

### Methodology

In order to give a comprehensive overview of the debate so far, we conducted a literature search to collect all publications that discuss ‘moral (bio)enhancement’ since the start of the debate in 2008. With assistance from a reference librarian, we selected suitable databases in bioethics. In September 2013, we searched these databases to find all publications that mention moral (bio)enhancement. For the specific search terms and strings per database, please consult Table 1. The results of these searches were downloaded to Endnote, and duplicates were removed.

**Table 1:** Search terms and strings

**Database**	**Search string**
**Embase**	(morality/de AND ('genetic enhancement'/de OR 'medical technology'/de OR 'neurosurgery'/de)) OR (((moral* OR virtue* OR virtuous OR biomedical* OR bio-medical) NEAR/6 (enhanc* OR bioenhanc* OR manipulat*))):ab,ti
**Medline OvidSP**	((morals/ OR Moral Development/ OR Virtues/) AND ("Genetic Engineering"/OR exp "Biomedical Enhancement"/OR "neurosurgery"/)) OR (((moral* OR virtue* OR virtuous OR biomedical* OR bio-medical) ADJ6 (enhanc* OR bioenhanc* OR manipulat*))).ab,ti.
**Web-of-science**	TS = (((moral* OR virtue* OR virtuous OR biomedical* OR bio-medical) NEAR/6 (enhanc* OR bioenhanc* OR manipulat*)))
**PsycINFO OvidSP**	((morality/OR Moral Development/OR Virtue/) AND ("Genetic Engineering"/OR "neurosurgery"/)) OR (((moral* OR virtue* OR virtuous OR biomedical* OR bio-medical) ADJ6 (enhanc* OR bioenhanc* OR manipulat*))).ab,ti.
**PubMed publisher**	(Moral enhanc*[tiab] OR Moral bioenhanc*[tiab]) NOT medline[sb]
**Scopus**	TITLE-ABS-KEY(((moral* OR biomedical* OR bio-medical) W/3 (enhanc* OR bioenhanc*)) AND (ethic* OR bioethic*))
**Google scholar**	"moral (enhancement|bioenhancement|enhancing)"|"moral bio enhancement"
**Scirus – preferred web/ProQuest**	"Moral enhancement" OR "Moral bioenhancement"

Based on title and abstract, we excluded all articles that are not directly related to moral bioenhancement. As the main aim of this paper is to provide an overview of the ethical reasons for and against moral bioenhancement in the debate so far, we included only those publications in which authors explicitly mention moral bioenhancement. We excluded from our analysis publications on the moral status of post-persons, unless there was an explicit reference to the debate on the desirability of moral bioenhancement. We also excluded those publications that discuss moral bioenhancement but were not written in English (N = 14). We discussed those publications that we were less sure about (N = 99) until consensus was reached. In April 2014 we repeated the exact same search, in order to retrieve all publications that were published in the intervening period (N = 22). All in all, 85 publications were included. For a schematic overview of the selection process, see Table 2 and Additional file 1.

**Table 2:** Selection of publications

**Database**	**Initial results**	**Results after deduplication**
**Embase**	1027	1008
**Medline OvidSP**	820	178
**Web-of-science**	1191	788
**Scopus**	449	261
**PsycINFO OvidSP**	427	253
**PubMed publisher**	17	10
**Google scholar**	192	142
**ProQuest**	75	58
**Scirus**	5	3
**Total**	4203	2701

We read the full-text of all articles and conducted a thorough thematic document analysis, in which we identified and coded each argument for or against moral bioenhancement mentioned in each publication. Based on this analysis, we formulated six broad clusters of arguments: arguments on the need for moral bioenhancement, on the possibility of attaining sufficient agreement on what moral bioenhancement should involve, on the status of current scientific research, on whether means and processes matter with respect to the desirability of moral bioenhancement, on the effects on the identity and the autonomy of the individual, and finally on the social effects of moral bioenhancement. This clustering was further refined and complemented on the basis of the analysis of all included publications, resulting in the final categories and subcategories that can be found in this article (see Table 3 for an overview of the arguments and sub-arguments we identified).

**Table 3:** Arguments for and against moral bioenhancement, presented in the reviewed literature

**Cluster**	**Argument**	**Description/background**	**Key articles**
**1. Why we (don’t) need moral bioenhancement**			
	There is scope for improvement	Almost by definition, each person can be/act/behave better. We therefore all have a moral duty/imperative/reasons to enhance ourselves. We have good reasons for wanting to better ourselves. Also: a duty to do the right thing.	[1,2,4-14]
	Human biological nature is defective	Humans are innately evil. Evil cannot be eradicated by socialization and education alone. Or: humans are innately good.	[2,12,15-27]
	Traditional means are (not) effective enough	Such as education, upbringing, socialization. These will only bring us so far. Or: they do suffice, are attractive and effective.	[2,15,17,19,21,26-36]
	Our only hope in averting major disaster	Avoidance of ultimate harm. Some of the world’s most important problems can be attributed to moral deficits of individuals. Or: those problems have other causes besides the moral deficits of individuals. Moral enhancement should accompany, or even precede/ prioritize over cognitive enhancement and scientific progress.	[1,2,8,15,17,19,21,24-29,34,36-46]
	Moral bioenhancement might reduce criminality	Promise of solving immoral and criminal acts. Or: warning that these are not necessarily the same.	[8,15,23,47]
**2. It will (not) be possible to reach consensus on what moral bioenhancement should purport**			
	No consensus on the mechanisms that comprise our moral psychology	The way we should interpret neurobiological findings.	[1,2,5,8,10,15,48-56]
	Behavioral changes alone are (not) enough	Emotions versus moral reasoning. Dependent on view on what is considered worthy of moral appraisal. Behavioral control, or: certain attitudes towards behavior are also necessary (they have cognitive content).	[2,5-8,11,12,15,17,23,29,31,32,34,44,46,48-51,54,55,57-67]
	Ethical systems and theories differ and often disagree	Subjectivity of/disagreement between main (substantive) moral theories. Individuals and cultures differ, there is moral pluralism. Possibility of being neutral between different conceptions of the good.	[1,2,4,8,10,12,13,15,16,23,24,29,31,48,49,52-55,63,64,67-69]
	(Im)possibility of considerable consensus	The question whether we can find a common ground, despite moral pluralism. Also: discussions on relativism/nihilism, objectivism.	[1,7,13-15,18,24,29,34,42,48,54,55,63,65,67,69-71]
	Situation- and role-dependency	Situation dependency of what counts as an improvement (morally). Different roles, assessments of situations. Weighing relevant reasons to act. One virtue can turn into a vice dependent on the situation.	[1,5,7,9,12,16,17,23,38,49,51,63,64,66,72-74]
	Human enhancement versus treating mental disorders	Enhancing humanity or treating mental disorders. Moral element in mental disorders.	[4,8,23,24,29,34,61,72,75,76]
**3. The feasibility of moral bioenhancement and the status of current scientific research**			
	Status of current scientific research	Further research is needed or, technological possibilities are already there.	[2,11,15,18,19,23,27,42,48,59,61,67,70,72,77]
	Complexity of our moral psychology/biology	Makes it doubtful that we will gain sufficient understanding.	[1,2,6,8,10,11,15,16,18,19,22,28,33-35,37,42,48,49,53,59,61-64,72-74,76-79]
		Is morality genetically/biologically determined? For example: are virtues and vices heritable? Is the core of our moral dispositions malleable by biomedical and genetic means? Danger of reductionism: we should not overlook the impact of the socio-cultural environment.	
	Unintended or undesirable side effects	Interventions have effects beyond the intended effects (also: bluntness of the instruments).	[5,7,8,11,12,16-19,22,23,29,30,34,35,42,46,50,51,60,62,63,69,75,77]
		A ‘baby and bathwater’ problem.	
		Moral bioenhancement might even lead to the opposite: not moral progress but moral decline.	
	Scientific rigor, standards	Research ethical questions about standards of good/sound science. Is scientific experimentation permissible, given that ‘lack of moral virtue’ is not a disease?	[30,36,48]
**4. Means and process of arriving at moral improvement matter ethically**			
	Other (non-biomedical) methods are preferable	Such as moral training, socialization or (self-) education. Taking a pill might seem ‘all too easy’ or too disconnected from ordinary human understanding. Are biomedical means intrinsically bad? Also: man is not supposed to play God.	[1,5-7,9-11,15-17,23,25,32,33,35,58,62,80]
	There is no principled difference between traditional and biomedical means	Results matter, the means less so.	[7,15,18,29,32,54,64,68,74]
		Perhaps the difference lies in the irrevocability/irreversibility of biomedical means.	
**5. Arguments related to the freedom, identity, and autonomy of the individual**			
	Moral bioenhancement might threaten the freedom of the individual	Moral bioenhancement might impair our freedom and diminish our freedom to act on bad motives. It might subvert moral agency.	[1,5,7-9,15,17,20,21,28,29,42,44,49,51,55,58-60,64,65,67,68,75,81,82]
	Moral bioenhancement might endanger our identity and autonomy	Questions about personal identity, and ‘true’ versus ‘brute’ self.	[1,4,8,9,30,33,51,57,64,67,68,75,78,81-84]
		Enhancer decides on outcome of moral bioenhancement (paternalism). Might compromise autonomous, informed choice.	
	Despite concerns about individual liberty and autonomy, a trade-off is justified	The advantages outweigh the disadvantages.	[1,5,20,21,29,42,45,60,67,68,74]
**6. Arguments related to social/ group effects and dynamics**			
	Moral bioenhancement benefits others	Unlike other types of enhancements (cognitive, cosmetic, sports). Or: who benefits? The individual or society as a whole?	[1,7,30,50,63,85]
	Moral bioenhancement might foster abuse	Moral bioenhancement might induce free-riding (e.g. prisoner’s dilemma). The virtuous exposed to exploitation by the vicious. It may lead to moral decline.	[1,5,8,9,14,15,22,31,33,42,48,56,61,63,64,66,76,79,86]
	Moral bioenhancement might undermine moral diversity and moral debate	It might diminish opportunities for ethical thinking/debate. Reasonable pluralism. Moral bioenhancement might generate social inequalities, elitism.	[4,14,23,31,47,48,53,54,61,63,71,74,76,79,85,86]
	Risks of utopian derailing	Progressive, well-intended, yet…	[5,9,11,16,18,30,31,33,63,64,87]
		Utopian.	
		Interventions will be used recklessly or overenthusiastically. Moral perfectionism.	
	Mandatory implementation or free/parental choice	State neutrality versus free choice. Danger of tyranny/discrimination.	[2,9,13,15,20,21,30,38,39,44,45,48,53,54,56,63,66,68-71,74,76,81,87]

We have conscientiously attempted to provide a neutral and comprehensive review of the existing arguments, by clearly separating the description of the arguments (see subsection ‘Arguments used in the debate’) from our critical appraisal of the arguments (see subsection ‘Critical appraisal of the current debate’).

### Arguments used in the debate

Table 3 provides an overview of the clusters of arguments and sub arguments we identified, as well as an overview of the authors addressing the specific argument. The arguments are formulated in a neutral way, and are almost always used by some to argue in favor of moral bioenhancement and by others to argue against it.

Below we will present the arguments we identified in the literature, organized in the following six clusters: (1) why we (don’t) need moral bioenhancement, (2) it will (not) be possible to reach consensus on what moral bioenhancement should purport, (3) the feasibility of moral bioenhancement and the status of current scientific research, (4) means and processes of arriving at moral improvement matter ethically, (5) arguments related to the freedom, identity and autonomy of the individual, and (6) arguments related to social/group effects and dynamics.

In the next section, we will describe these arguments in greater detail by summarizing these six clusters and their components. Given the richness of the publications we studied, it will not be possible to take account of all the arguments in great detail. However, in the following paragraphs we hope to sketch the outlines of the discussions held so far and to provide an overview of the main arguments identified under clusters one through six. Relevant subthemes will be discussed under each cluster.

### 1. Why We (Don’t) Need Moral Bioenhancement

The arguments gathered under this first cluster address the question as to why (or whether) we in fact need moral bioenhancement. What kinds of problems we hope it would eradicate, what its advantages are compared to other methods, and how it relates to traditional methods of moral betterment. It is clear that most proponents of moral bioenhancement feel the need to offer some story on why there is in fact an urgent need for it. Opponents or sceptics may doubt whether we need moral bioenhancement at all.

#### There is scope for improvement

Almost by definition, most if not all people would benefit from an improvement in their moral character. Different authors vary, however, with respect to the kind of changes they would like to see implemented: changes in moral behavior, will-power, or moral agency and insight.

Because the moral character of most people is suboptimal (or even defective by nature), every person has good reasons to morally better herself. The general argument holds that we have a moral duty to enhance ourselves, and that if we need moral bioenhancement to reach this goal, we should consider it: “it is not that taking medicine is intrinsically moral or immoral, it is that a human subject can use medication as a means to assist them towards a moral end: reducing future harm. Such a person exhibits altruism” ([57]: 180). The only right attitudes towards one’s own bad motives and impediments are non-acceptance and a desire for self-change, Thomas Douglas ([1]: 235) maintains.

Where Douglas [1] presents the recognition that there is room for improvement as an argument for moral bioenhancement, others are of the opinion that although we can agree that the world we live in now is far from optimal, it is not clear why this would be a reason in favor of moral bioenhancement. For example, according to Nicholas Agar: “We don't need superior moral vision to understand that poverty, climate change, and terrorism are bad things. (…) We do need enhanced effort and perhaps enhanced nonmoral powers to fix poverty, climate change, and terrorism but we don't need enhanced moral vision to recognize that they need fixing” ([4]: 75).

#### Human biological nature is defective

In defense of the need for moral bioenhancement to morally better ourselves, Ingmar Persson and Julian Savulescu argue that there is a fundamental mismatch between our moral psychology and today’s conditions of human life ([28]: 124). Because human moral psychology evolved in conditions that are radically different from those in today’s world, we should alter human moral psychology by biomedical and genetic means, they argue:

People encode the race of each individual they encounter, and do so via computational processes that appear to be both automatic and mandatory. (…) If genetic and biomedical means of enhancement could counter such natural tendencies, they could have a crucial role to play in improving our moral character, that could complement traditional social and educational means of moral enhancement ([2]: 168).

Others add that besides being ill equipped for today’s conditions of human life, human beings are innately evil to a greater or lesser extent. Wickedness is an indispensable part of human nature. If we want to eradicate evil, we have to alter these immoral innate (biologically determined) tendencies of human beings. Socialization, upbringing and education will bring us only so far. According to Mark Walker, precisely because humans are evil by nature, we need biomedical interventions in order to effectively alter human nature for the better: “For sure, it may be possible to minimize some contemporary evil through better socialization, but it will never be possible to eliminate it so long as human nature remains unaltered” ([15]: 29).

On the other hand, Robert Sprinkle argues that the observation that evil may not be an eliminable feature of the human condition should temper our hopes regarding the possibility of effectively addressing all forms of evil, not raise them: “I, for one, never held such a hope” ([16]: 89). John Harris turns the ‘humans are evil by (biological) nature’-argument around and argues that there is an inborn human goodness: “We have certainly evolved to have a vigorous sense of justice and right, that is, with a virtuous sense of morality” ([17]: 104).

#### Traditional means are (not) effective enough

In addition to stating the need for moral bioenhancement due to our defective moral nature, some authors argue that traditional means are ill-equipped or less effective as compared to biological and/or genetic means. As mentioned by David DeGrazia, surely, we already have at our disposal many different means of enhancing our moral capacities: methods such as “explicit moral instruction, mentoring, socialization, carefully designed public policies, consciousness-raising groups, literature and other media that encourage moral reflection, and individual efforts at improvement” ([29]: 361). However, Persson and Savulescu argue that these means are not nearly effective enough to help us counter the great evils of our time: “Biomedical and genetic means may be much more effective in terms of both how thoroughly and quickly they could improve everyone in need of improvement” ([2]: 168).

Others, for example John Harris and Jamie Bronstein, feel that this line of argument wrongly minimizes the moral progress that has been made through those tried and tested traditional methods ([17]: 104, [30]: 86), and argue that these methods still offer many possibilities for moral improvement.

#### Our only hope in averting major disaster

On top of the need for effective interventions to morally better ourselves, *urgency* is another critical factor that is addressed in the debate. If we succeed in (biomedically) enhancing people’s cognitive abilities, some argue, it is of paramount importance to also – or even first – enhance their moral abilities due to the risks that cognitively enhanced human beings may pose to others. In today’s technologically advanced world, Persson and Savulescu argue that a “morally corrupt minority” ([2]: 163) is increasingly able to inflict major disaster on the majority. Moral bioenhancement might be our only hope of engaging with other major challenges as well. According to DeGrazia:

The status quo is deeply problematic because there is such an abundance of immoral behavior, with devastating consequences, and serious risk of worse to come. (…) In addition to these harms and injustices, there is the threat of truly massive harm. (…) It is increasingly possible for a small number of individuals to acquire the technical capability of inflicting terrible harm ([29]: 362).

Harris, however, argues that we should instead embrace cognitive enhancements, as they are our best prospect of self-defense against disaster ([17]: 110). Adam Carter and Emma Gordon argue that because cognitive and moral enhancements are principally interconnected, we should consider potential enhancements “*outwith* any essential reference to a moral/cognitive conceptual dichotomy” ([37]: 8).

David Wasserman questions whether all of these evils can be attributed to individual moral defects, and warns us not to underestimate the role of defective institutions ([38]: 375).

#### Moral bioenhancement might reduce criminality

Last but not least, some authors such as Walker [15] suggest that moral bioenhancement could achieve a significant reduction in ‘evil’ , referring to criminal behavior such as rape, murder, torture and so on. Others, for example Thom Brooks, warn us that immorality and illegal behavior do not necessarily coincide:

Morality and law are imperfectly linked at best. First, not all immorality is illegal. Lying is widely regarded as immoral, but not all lying is criminal. (…) Second, not all illegality is immoral. Drug offenses are widely incorporated in most legal systems, but it is unclear at best whether cannabis use is intrinsically immoral. ([47]: 29)

Because the project of moral bioenhancement and the project of reducing criminality are not necessarily the same, it is argued that we should be careful in suggesting that moral bioenhancement might indeed reduce criminality and using this as an argument for moral bioenhancement.

### 2. It Will (Not) Be Possible to Reach Consensus On What Moral Bioenhancement Should Purport

In this second cluster we discuss arguments that address the many different kinds of disagreement that influence the different proposals on the way moral bioenhancement should take shape. Opponents of moral bioenhancement think that because of these allegedly fundamental disagreements, moral bioenhancement is a problematic endeavor. Proponents, however, argue that sufficient consensus is possible, and that these differences need not necessarily jeopardize the project of moral bioenhancement.

#### No consensus on the mechanisms that comprise our moral psychology

The first issue that fuels disagreement on the way moral bioenhancement should take place relates to our limited knowledge concerning our moral psychology. Moreover, according to Persson and Savulescu, how we should interpret and understand findings of moral psychology is not straightforward, and influenced by our preferred view on what constitutes morality: “what morality is, or of what it is to be moral” ([2]: 168). Agar warns us that:

The absence of a consensus upon the mechanisms of morality could prevent any agreement that a proposed moral enhancer could really be enhancing morality, whatever else it may be doing. This skepticism is not the fault of the behavioral and brains sciences, but our own, for failing to agree about which cognitive processes are genuinely relevant to what we want to call morality and moral agency ([48]: 5).

#### Behavioral changes alone are (not) enough

Even if a consensus would exist on the mechanisms of morality and how to achieve more moral behavior, several authors question whether it is enough for any moral bioenhancement intervention to have effect on behavior, but not necessarily on other aspects of morality, such as moral reasoning, moral insight, or moral will. Do behavior control interventions constitute moral enhancement or does moral enhancement require an accompanying change in moral agency?

According to Douglas [1,5,6], reducing an individual’s tendencies towards violent aggression *directly*, without using cognitive means such as persuasion or deliberation (and assuming this would effectively lead to less immoral behavior), would count as moral enhancement. Harris [17,31,58-60], however, insists that without concurrent changes in a person’s moral reasoning, these changes would not amount to moral enhancement at all:

We will, I believe, always need to use moral reasoning to act as a guide to our emotions and as a way of checking that we are having appropriate feelings in appropriate circumstances and for appropriate objects. If the good involves feeling the right way, how do we know that we are feeling the right way? ([58]: 172)

Fabrice Jotterand also argues that “the emphasis on the control of moral emotions appears reductive and one-sided in the sense that it conflates moral reasoning (as practical reasoning) with moral psychology (how moral reasoning acts on one’s motivational/emotional states)” ([49]: 5).

Bernard Baertschi argues that much of the disagreement between Douglas and Harris can be attributed to their different preferred meta-ethical positions ([50]: 66-67). Harris adheres to a rationalist conception of ethics, according to which emotions should only be acted upon through cognitive means, and, as described by Baertschi, “reason furnishes the only genuine moral motives” ([50]: 66). Douglas however appears to be a sentimentalist, Baertschi argues, i.e. espousing a view on ethics according to which having the right feeling matters. Their different conceptions of what morality *is* influence their different assessments of whether moral bioenhancement might be effective. Whereas Douglas thinks that direct modulation of emotions is effective (and permissible), Harris denies that direct modulation of emotions even amounts to moral enhancement at all.

#### Ethical systems and theories differ and often disagree

The wide variety of substantive moral principles that characterizes debates between ethical theorists, also hampers agreement on what would constitute a moral enhancement. John Shook provides the following example:

Suppose a brain modification transforms a person into someone who now takes the moral deed to be the one maximizing the welfare of all. (…) most utilitarians would soon find fault with this new utilitarian’s concrete moral judgments, just as they find fault with each other’s. And many deontologists would simply deny that this fresh utilitarian has received moral enhancement at all ([48]: 4).

Nevertheless, as DeGrazia argues, in one way or another, every program for moral bioenhancement needs to make explicit what it considers to be a moral improvement, based on what kind of principles or theory. Differences between various moral theories are not necessarily purely theoretical, and may lead to different normative judgments concerning a variety of moral dilemmas (e.g. abortion, the death penalty, and euthanasia) ([29]: 363).

Next to disagreement between the major ethical theories, DeGrazia reminds us that individual (groups of) people hold diverse and often conflicting moral outlooks. They differ greatly with respect to the values they adhere to: politically conservative or progressive values for example ([29]: 363).

#### (Im)possibility of considerable consensus

In addition to the fact that a consensus is currently lacking, some authors view ethical standards as “arbitrary products of cultural history”, as Larry Arnhart wonders with regard to Walker’s writings ([70]: 81). Or, given a pluralistic reality, is it possible to be neutral with respect to wide-ranging outlooks and ethical systems? Persson and Savulescu, for instance, expect that their proposal for the core of our moral disposition – consisting of altruism, a sense of justice or fairness, and empathy – will be shared by many ([2]: 168-169). They think that despite the deep disagreements between different accounts of right action, some sort of actions (“the willingness to sacrifice one’s own interests for the benefit of others”, for example) will be viewed as “a moral enhancement, on any account of morality” ([68]: 5-6).

Walker also cautions against overemphasizing the differences, and points to significant overlap between different lists of virtues ([15]: 35). DeGrazia proposes that we “stick to improvements that represent points of overlapping consensus among competing, reasonable moral perspectives” ([29]: 364). Moreover, Filippo Sio and colleagues claim that:

As in the cases of cognitive enhancement and social progress, reference to some objective standards is also necessary to make the concept of moral enhancement coherent ([71]: 15).

#### Situation- and role-dependency

In addition to different views on morality and moral behavior, situation- and role-dependency further add to the confusion concerning what should count as a moral enhancement. What should count as an improvement is highly dependent on the specific context and the roles performed in that situation (e.g. detached surgeons to remove brain tumors, impartial judges to administer justice). Wasserman asserts that even slight moral improvements will vary according to the role and context in which these are brought about [38].

Sprinkle approvingly cites Aristotle’s assertion that “traits virtuous in moderation might be vices in absence or excess” ([16]: 89). Moreover, Markus Christen and Darcia Narvaez argue that “moral character cannot emerge from a short-term intervention, but, as Aristotle advised, must be shaped with mentoring through multiple situations over time” ([32]: 26). Moreover, Sarah Chan and John Harris refer to a situation in which “serotonin-induced aversion to inflicting direct harm” might have stopped passengers from forcefully stopping a would-be hijacker ([51]: 131). In situations like these, aggression can be a good thing, although it is clearly bad in others. From this it follows that even enhancing traits that everyone would agree to be 'good’, may still not result in an overall, all-purpose, moral enhancement. However, such criticisms on moral bio-enhancement fail to consider, according to Kahane and Savulescu, that this is “not at all an argument against enhancement but, rather, an argument for more precisely fine-tuned enhancement” ([12]: in press). For example, by making biomedical interventions sensitive to certain contexts, but not to others.

#### Human enhancement versus treating mental disorders

Some authors, such as Agar [72] and Jotterand [61], highlight the difference between enhancing moral capacities of individuals “beyond human norms” ([72]: 369) and treating mental disorders that may or may not contain “an inherent moral element” ([61]: 1). Dorothee Horstkötter and colleagues for example argue that “if there is a health problem, medical treatment is the reasonable reaction, while enhancement, either moral or otherwise, does not arise” ([75]: 27).

### 3. The Feasibility of Moral Bioenhancement and the Status of Current Scientific Research

Under the third cluster, we discuss arguments based on the alleged feasibility of proposals for moral bioenhancement. Whereas proponents of moral bioenhancement are optimistic about the status of current scientific research, opponents warn us that the complexity of our moral psychology and biology make it doubtful that we will be able to develop effective interventions.

#### Status of current scientific research

Current scientific developments give rise to both high hopes and substantial skepticism. DeGrazia, for example, rather optimistically lists research that may further the science of moral bioenhancement, ranging from the use of selective serotonin reuptake inhibitors as a means to being less inclined to assault people, to deep brain stimulation as a way to reduce aggression ([29]: 361-362). Walker sees no technological reasons why the pre-implantation sorting of embryos that is presently used to screen for genetic diseases could not be used for selecting virtues ([15]: 31).

Molly Crockett warns us against overstating the conclusions of single studies, and asserts that science in this field is “in its infancy” ([77]: 370). With respect to genetic engineering Arnhart observes that clear examples of “specific genetic linkages to virtue that could be altered” are presently unknown ([70]: 79).

Persson and Savulescu admit that their proposals for moral bioenhancement are mostly based on hypothetical scenarios, and treatment at this moment is only possible to a very small extent: “A lot more scientific research is needed before we can be made more altruistic or just by suitable drugs or surgery, or genetic manipulation” ([2]: 172)

Although William Kabasenche thinks that human moral psychology is highly complex and therefore that it is difficult to ‘engineer’ virtues and vices, he feels that these studies “give us important insights into what embodied virtues might look like, and they suggest that we should take our embodied nature seriously” ([62]: 20).

#### Complexity of our moral psychology/biology

Regardless of an optimistic or pessimistic view on the status of current scientific research, it is also argued that character traits, such as those involved in human morality, are highly complex, and therefore that moral bioenhancement is probably not feasible. These reservations are not only expressed with respect to the manipulation of neurotransmitters, but also with respect to the possibilities of genetically engineering virtues.

Arnhart doubts that we are anywhere near having found the “generic correlates of virtue that are clear, strong, and manipulable” [70]: 80]. Walker however thinks that, given the fact that much progress has been made in the behavioral genetics of schizophrenia, this field can show the way forward to investigating the possibilities of a comparable behavioral genetics of virtue: “After all, genes for schizophrenia are polygenetic and show intensity of expression and gene-environment interactions” ([18]: 91-92). Douglas also feels that there are some elements of our moral psychology that we are beginning to understand to such a degree that manipulating them is possible: "it does not seem unreasonable to suppose that moral enhancement technologies which operate on relatively simple emotional drives could be developed in the medium term" ([1]: 233).

Some authors, for example Robert Sparrow, voice concerns about reductionism, i.e. “the claim that whether an individual is a (morally) good person is a function of that person’s neurochemistry and/or that person’s genetics” ([63]: 27). Others, such as Hans-Joerg Ehni, Diana Aurenque [33] and Chris Zarpentine [19], warn us not to underestimate the importance of societal and cultural influences.

#### Unintended or undesirable side effects

Given the complexity of our moral psychology and biology, can we hope to influence it without also altering other crucial processes? Crockett [77] warns against the unintended, and possibly undesirable, consequences of altering the function of a specific neurotransmitter, beyond the desired effects on moral behavior. Karim Jebari [7], for example, discusses findings that suggest that enhancing empathy may render individuals less fair and more partial rather than less fair and more impartial. Agar provides the following example:

What we recognize as the correct pattern of judgment strikes a particular balance between the call of empathy and the appeal of moral reasoning. (…) Unbalanced enhancement of empathy is likely to disrupt what we view as the morally correct trade-off between benefits conferred on those to whom we are socially bonded and costs experienced by those to whom we are not socially bonded. It tends to reinforce our tendency to endorse solutions that inflict suffering on strangers to protect our nearest and dearest from less significant suffering ([34]: 2).

In the case of genetic engineering, many more systems than just the targeted virtue or vice might be effected. For example, Bronstein asks: “What happens if selecting for virtuous genes also increases the likelihood of cancer, diabetes, heart disease, or even shyness or depression?” ([30]: 85).

#### Scientific rigor, standards

In addition to the scientific and philosophical uncertainties regarding our moral psychology and biology, some authors touch upon the issue of future scientific experimentation with respect to moral bioenhancement. Bronstein asks whether medical experimentation is permissible, given the fact that immorality is not a disease: "Walker's project design may also violate one of the great principles of human experimentation: that medical experimentation approved by proxy on behalf of those who cannot consent must benefit the patient. Lack of moral virtue is perhaps suboptimal, but we have not yet classified it as a disease" ([30]: 85). Bronstein further observes that, in the case of Walker’s Genetic Virtue Program, many questions can be asked with respect to the research design:

Will genetically modified humans be raised in controlled environments so that they can be more easily observed and exposed to uniform socialization? If not, how can we know whether these particular humans are indeed more virtuous, and that their virtue is indeed genetic? ([30]: 86)

### 4. Means and Processes of Arriving at Moral Improvement Matter Ethically

Under the fourth cluster, we discuss arguments that explore the question as to whether the difference between biomedical and non-biomedical means matters ethically.

#### Other (non-biomedical) means are preferable

From an intrinsic perspective, it is often considered whether it would be better, more praiseworthy, or more authentic, if a person betters herself without resorting to biomedical means? Or as Douglas puts it: is it the case that adopting “biomedical means to moral enhancement is objectionable not just relative to other alternative means, but in an absolute sense” ([1]: 236)? Sprinkle thinks that even if a safe and “convincingly enduring” intervention would become available, people would likely prefer a non-genetic remedy ([16]: 89). This argument implies that we have intrinsic reasons, such as authenticity and personal identity, to reject biomedical interventions in favor of traditional means for moral enhancement. It also assumes that there is a principled difference between biomedical and traditional, non-biomedical means.

Others argue that biomedical and non-biomedical means will be used in concert, rather than separately. According to Douglas [1], we will likely regard biomedical enhancement and self-education as complementary and are likely to reinforce the desire for both by initially engaging in one or the other. Moreover, according to Kabasenche, “none of us achieve any measure of success in moral formation without significant assistance from others. If authentic moral formation is something you do completely by yourself, none of us has done it" ([62]: 20).

#### There is no principled difference between traditional and biomedical means

Alternatively, some authors argue that there is no principled difference between using traditional and biomedical means to morally better oneself or others. DeGrazia, for example, argues that (many of the) arguments against biomedical means also apply to traditional, non-biomedical means: “one should not inculcate moral values that are wrong, so how can a parent be sure that she or he is justified in providing a particular type of moral instruction? Also facing this challenge are public school teachers who attempt to inculcate in students certain moral virtues such as civility, respect for differences and concern for the poor” ([29]: 363).

Likewise, according to Walker’s ‘companions in innocence’ line of reasoning, any principled argument given against biomedical means for moral enhancement, such as those involved in his Genetic Virtue Program, equally applies to socialization and education efforts: “If the [Genetic Virtue Program] is wrong in attempting to promote virtue as a means of making people morally better, then much current socialization and education is mistaken as well” ([15]: 35-36).

Sparrow however maintains that a significant disanalogy exists between traditional means of moral improvement and the biological manipulation of behavior and motivation. Whereas education is characterized by “a fundamental moral equality between educator and educated”, biomedical interventions to reshape the agency of others “involve a subject acting towards an object and as such are fundamentally structured by a profound inequality” ([64]: 26).

### 5. Arguments Related to the Freedom, Identity, and Autonomy of the Individual

Under cluster five, we discuss various arguments regarding the question as to whether moral bioenhancement would limit the individual in his or her opportunities to freely choose his or her behavior? We focus on concerns related to individual liberty and autonomy.

#### Moral bioenhancement might threaten the freedom of the individual

The concept of freedom takes a central role in the moral enhancement debate, with Harris being one of its most ardent defenders. He is of the opinion that individual liberty is of utmost importance, and should take priority over all other good ends that we might pursue. He explicitly opposes “any measures that make the freedom to do immoral things impossible, rather than simply making the doing of them wrong and giving us moral, legal and prudential reasons to refrain” ([17]: 105). William Simkulet argues that “when one is forced against one’s will to do as the virtuous person in one’s place would freely do” we should speak of moral compulsion instead of moral enhancement ([65]: 17).

Some authors, such as Jebari [7] and Birgit Beck [8], call for conceptual clarification of a suitable concept of freedom in the debate on moral enhancement: what kind of freedom is at stake?

#### Moral bioenhancement might endanger our identity and autonomy

Related to the worries concerning individual liberty, concerns are voiced that moral bioenhancement could pose a threat to our true, autonomous self. Douglas argues that the counter-moral emotions that would be altered are at best part of a person’s ‘brute’ self, and thus enhancement would be “allowing his true self greater freedom.” Our true or authentic self refers to our internal characteristics whereas our brute self refers to anything that is external to this ([1]: 240). Certain enhancements could alter our brute self in such a way that it constrains our true self, thereby threatening our freedom and autonomy. At the same time, others argue that we should not overestimate an individual’s capacity for full autonomous behavior as exemplified by his/her ‘true self’. For example, Russell Blackford [78] warns against attributing to ourselves a “spooky kind of autonomy all the way down” that does not exist in the real world.

Some authors also worry about possible changes of identity as a result of moral bioenhancement. Douglas distinguishes a loss of identity in a strong sense, in which an individual would, post-enhancement, be a completely different person, and a weak sense, in that moral bioenhancement would change some of her most fundamental psychological characteristics ([1]: 239). Yet Douglas stresses that we only have reason to preserve those psychological characteristics that have positive value for the individual in question. Whereas Douglas emphasizes that the individual is free to choose, others such as Agar [4] are less clear on how the positive value of particular psychological characteristics is to be determined: on the basis of an individual’s own judgment, on someone else’s judgment (e.g. within a criminal justice contexts), or on a specific moral theory?

Given the large diversity of potential moral enhancements, moral enhancement interventions will inevitably prioritize some moral values or character traits over others. This, some argue, will place the person who undergoes the intervention at the mercy of the person performing the intervention: “Someone who has been subjected to moral enhancement is likely to have a reduced sensitivity to moral reasons rejected by his or her enhancer" ([4]: 75).

#### Despite concerns about individual liberty and autonomy, a trade-off is justified

Acknowledging that moral bioenhancement might indeed negatively impact the freedom, autonomy, or identity of an individual, should this stop us from pursuing moral bioenhancement? Some authors, for example Douglas [5] and DeGrazia [29], stress that although such a loss might be regrettable, if it is compensated by an increase of some other good, the loss can be justified. DeGrazia, for example, maintains that “we should not exaggerate the value of freedom. After all, moral behavior itself, the end product, is also extremely important—independently of how free it is” ([29]: 367, see also Persson and Savulescu, [20]: 252). Savulescu and Persson claim that:

We are not free to commit serious crime even now – the laws prohibits it on pain of punishment. What we weren’t free to do, the God Machine makes strictly impossible. If this is a loss, it would be outweighed by the fact that there are no victims suffering from serious crimes ([88]: 13).

Bronstein is not convinced however that this trade-off is justified: "one might ask whether the goal of moral perfection is worth the trade-off for human autonomy on a large scale” ([30]: 86).

### 6. Arguments Related to Social/Group Effects and Dynamics

Finally, under the sixth cluster, we discuss arguments that consider possible societal and group effects of moral bioenhancement. Would moral bioenhancement foster abuse? Should its use be mandatory, and who should decide? Is there overconfidence in the possibilities of biomedical solutions?

#### Moral bioenhancement benefits others

To start, Douglas situates questions about the desirability of moral bioenhancement in the context of the wider enhancement debate, and argues that the fact that (unlike other enhancements) moral enhancement primarily benefits others, neutralizes many of the objections often raised in the broader enhancement debate: “moral enhancement[s] could not easily be criticized on the ground that their use by some would disadvantage others” ([1]: 230).

Bronstein however turns this argument around, and criticizes Walker’s Genetic Virtue Project precisely because it appears to prioritize the benefits to others over the benefits to the agent ([30]: 85). He worries that in the design of the project, the interests of society will take priority over the interests of the individual.

Walker [15] raises the worry that the biologically unenhanced might be discriminated against in favor of the biologically enhanced, but immediately adds that it is not clear whether the same incentive for discrimination would arise in cases of moral enhancement as can be expected in cases of physical and/or cognitive enhancement.

#### Moral bioenhancement might foster abuse

Another concern that is put forward, is the fear that an altered ratio of moral people to immoral people might give rise to free-riding: the few morally unenhanced might more easily take advantage of the good intentions of the many morally enhanced. This dynamic might be visible not only between groups of individuals, but between countries as well, Shook suggests: “Depictions of entire societies or a whole planet undergoing empathetic moral enhancement will remain utopian fantasies. One country after another will decline moral enhancement until the “worse” countries have done it, and each country would want their neighbors to go first” ([48]: 11).

Walker however thinks that this worry underestimates the resilience of the morally enhanced: “it seems to assume that the virtuous are meek or compliant" ([15]: 42-43).

#### Moral bioenhancement might undermine moral diversity and moral debate

Moral enhancement also raises questions with respect to which moral views or paradigms may benefit at the expense of others, and whether this may lead to a diminished diversity of views. Brooks argues that:

The question is not only whether moral enhancement might lead to only one moral judgment, but also whether moral enhancement might benefit some reasonable moral, philosophical, and religious doctrines over others. If not all reasonable doctrines may benefit equally, then moral enhancement might violate the equality between citizens and fail to respect the reasonable pluralism that exists ([47]: 29).

This may hamper the quality of political and social debate: Shook worries that moral enhancers “could diminish opportunity, capacity, and responsibility for serious ethical thinking” ([48]: 8) Moreover, in a future dystopia, moral enhancements may be regarded as suitable quick fixes in case of moral ambiguities and dilemmas, thereby further reducing valuable opportunities for serious ethical reflection: “individuals thinking too hard about moral ambiguities and dilemmas are told that they simply need their enhancers adjusted" ([48]: 8).

Sparrow [63] worries that the morally enhanced would gain important advantages, for example by their exclusive participation in social and political institutions, thereby generating or intensifying social inequalities.

#### Risks of utopian derailing

Related to the risk of disrespect for reasonable pluralism, some authors express the worry that the promises and high hopes of moral bioenhancement projects (for example Walker’s [15] proposal for a Genetic Virtue Project) will repeat many of the mistakes (such as mass regimentation and loss of autonomy) of what Bronstein calls ‘High Modernist planning’: “Walker's plan features all of the confidence, and many of the other signs, of High Modernist planning. The project that he proposes is transnational in scope; it seeks to transcend, or one might even say ignore, current political realities. Its emphasis is on the future, and it is enlivened by the discourse of the good” ([30]: 86).

Sprinkle recalls the excesses of the eugenics movement: “Walker's work should not be turned wrong-side-out. But neither can the lessons of the Eugenics Movement be taken as safely learned long ago. It was avowed to be a progressive movement, a product of progressive thought and an instrument for progressive action” ([16]: 89).

#### Mandatory implementation or free/parental choice

Despite the many reservations described above, if we assume that safe and effective moral bioenhancement would become available, would it be justifiable to make its use mandatory – for all, or for specific target groups? First, Bronstein raises doubts that many people will voluntarily seek moral bioenhancement: “how large is the distance between explaining what is good for you and imposing what we know to be good for you? (…) Genetic virtue is an idea that ought not to be imposed on an unwilling public—and seems unlikely to find a willing public” ([30]: 86).

Persson and Savulescu however argue that an unwilling public should not stop the program, and that safe, effective enhancements should be compulsory: “If safe moral enhancements are ever developed, there are strong reasons to believe that their use should be obligatory, like education or fluoride in the water, since those who should take them are least likely to be inclined to use them” ([2]: 174). Rakić [21,39] however argues that making moral bioenhancement obligatory would deprive us of an essential part of our human existence, that is, the freedom “for us acting *intentionally* in a morally appropriate manner” ([21]: 248)], and therefore advocates voluntary moral enhancement.

A specific version of this question arises with respect to children. Should parents be the ones to decide whether their children should undergo an intervention, or whether their future offspring should be genetically selected within the framework of Walker’s [15,18] Genetic Virtue Project? Walker himself favors “some hybrid or conditional option to mediate between the state-mandated versus liberal (parental choice) implementation” in which parents are free to choose which virtuous characteristics they would like to see enhanced, but in which they are not free to choose the associated vices ([15]: 43). Arnhart wonders who will be responsible for setting and enforcing the standards for these virtues and vices, and fears the “threat of tyranny – either the tyranny of a few or the tyranny of the majority” ([70]: 80-81).

### Critical appraisal of the current debate

The debate on moral bioenhancement is a fairly recent phenomenon. Douglas, in the first article on the subject in 2008, discusses moral bioenhancement as a theoretical possibility in the context of the discussion on the permissibility of enhancement in general. Persson and Savulescu [2] first discuss the possibility of moral bioenhancement in the context of the rapid developments in the field of cognitive enhancement. Although they are doubtful as to whether moral bioenhancement is in fact feasible in the foreseeable future, they nevertheless call for intensified research efforts because they see moral bioenhancement as the only possible solution for a number of pressing problems. Later on, the debate moved on to fundamental philosophical questions about what human morality involves, and whether or not we would be able to reach enough consensus to transcend our current pluralistic moral reality. In what follows, we critically assess the arguments discussed under the six clusters presented above, and identify those issues and concerns that have been neglected so far. We identified four topics of concern: (1) the distinction between treatment and enhancement; (2) an overestimation of the possibilities/feasibility of moral bioenhancement; (3) insufficient attention to side-effects, risks and safety; and (4) identity changes.

### The distinction between treatment and enhancement

There has been surprisingly little discussion concerning the criteria we should use to identify specific interventions as moral enhancement rather than as therapy or moral education. In the debate so far, it remains unclear whether ‘moral enhancement’ should be taken to include treating those with a pathological lack of certain moral capacities. Horskötter and colleagues argue that those interventions aimed at restoring normal moral functioning in subjects whose moral functioning is somehow pathologically impaired, should be called medical treatment, rather than enhancement ([75]: 27). Agar is one of the few authors in the debate who do make this distinction. He claims that targeted interventions aimed at “therapeutic ends” can possibly amend specific deficiencies, but that these same interventions “can produce unbalanced excesses when used to enhance beyond human norms” ([72]: 369). Other authors, for example Douglas [1] and DeGrazia [29], appear to use examples of general moral enhancement and specific mental pathologies, such as psychopathy and antisocial traits (e.g. violent aggression), interchangeably.

Although some, for example DeGrazia [29], reject the distinction between treatment and enhancement, and while it is clear that the exact boundaries will of course always be up for dispute, we believe that distinguishing between treatment of those with pathologies and enhancement of normal people will greatly benefit the debate. Shifting the focus to treatment of pathological deficiencies in morality raises new and interesting questions that are different from the ones raised in the debate on moral enhancement for normal people. What is to be considered normal moral functioning, and who is to determine whether a subject functions ‘normally’? Should subjects who lack certain capacities or who show ‘abnormal’ moral functioning be considered to have a disease or disorder? In other words: when should diminished moral functioning or immoral behavior be considered to be pathological? How should society deal with common moral deficiencies? As mentioned above, Persson and Savulescu [2] argue that safe and effective moral enhancement should be compulsory since those individuals that need them will be least willing and/or likely to use them. Such safe and effective moral bio-enhancements are not available at present and difficult questions remain to be answered. This brings us to our final remark regarding the distinction between treatment and enhancement: more debate is urgently needed on what would be the right kind of response towards those who behave immorally due to pathological deficiencies of their moral capacities: treatment or punishment?

### Overestimation of the possibilities/feasibility of moral bioenhancement

Although some authors, such as Bronstein [30] and Sprinkle [16], are very cautious and even skeptical, many if not most authors in the current debate voice an overwhelming enthusiasm concerning the feasibility and future applications of moral bioenhancement. However, based on the empirical possibilities available today and in the near future, this enthusiasm seems somewhat misguided. The lack of scientific scrutiny is particularly striking when the possibility of genetic screening and modification to morally enhance individuals and potentially reduce criminal behavior is put forward (e.g. Walker’s Genetic Virtue Project). Although genetic findings may improve our understanding of the risk factors associated with criminal behavior, we are far from identifying genetic risk factors for crime that could predict with reasonable certainty which individuals are at greater risk of engaging in criminal behavior. There is no one-to-one relationship between biological factors and criminal behavior. Indeed, depending on the environmental context, many individuals that carry biological risk factors for such behavior will not develop it, while others who do not show these risk factors might [89-91]. Genetic modification that could lead to reliable moral enhancement is extremely far removed from our present-day knowledge and capacities, and it is doubtful whether it will ever be successfully achieved.

Morality and moral behavior are associated with so many different areas of the brain, that it has been claimed that morality is everywhere and maybe nowhere in the brain [92]. Offenders with impaired moral decision-making, such as individuals with antisocial personality disorder, show overlapping abnormalities in several of these brain areas. Morality and moral behavior are very complex human traits and this is reflected at the developmental, experiential and neuroanatomical level, and most likely at the genetic level as well. It is misguided to think that we will be able to identify single genes or a single combination of genes that underlie morality and moral behavior. As most psychiatric and personality disorders are polygenic (i.e. involve a set of genes) and genetically heterogeneous (i.e. different sets of genes underlie the same diagnosis), it is highly likely that complex cognitive-emotional processes such as morality and (im)moral behavior are similarly associated with a myriad of genes and substantial genetic heterogeneity among different individuals. Similarly, not all antisocial individuals show the same biological deficits and a wide range of biological and environmental factors may contribute to antisocial behavior in a variety of ways [90].

Other potential interventions for moral enhancement could be neurofeedback, transcranial brain stimulation (e.g. magnetic stimulation, and direct current stimulation), electrical stimulation of the brain via electrode implants (e.g. deep brain stimulation or DBS), and neuron replacement therapy (currently investigational). Although some of these neurotechnologies have reached the stage of demonstrated clinical effectiveness for certain disorders (e.g. neurofeedback for ADHD, TMS for depression, and DBS for Parkinson’s), few have reached that level regarding phenotypic traits that likely contribute to (im)moral behavior. Nevertheless, preliminary studies indicate that transcranial brain stimulation might be effective for addiction, isolated case-studies exist where electrical brain stimulation via electrode implants is applied for chronic aggression or addiction, and preliminary experimental research suggests that functional magnetic resonance imaging neurofeedback could hold some potential for addressing addictions, antisocial personality disorder, psychopathy and sexual disorders. Improving ‘normal’ moral traits or behavior with such means is even further away from being practically feasible at present.

The idea of reliably bioengineering complex cognitive-emotional processes such as altruism or virtues is not feasible in the near future and it is highly unlikely even in the distant future. Because so many different biological and environmental factors influence an individual’s (im)moral behavior, we are convinced that biomedical means alone will not suffice for moral enhancement. Hence, the debate on moral enhancement should take as its starting point a combined approach in which traditional methods and emerging biomedical methods are used in tandem.

### Little attention to side-effects, risks and safety

Except for psychopharmacological or hormonal treatments, few actual or potential interventions for moral bio-enhancement have been discussed in the moral enhancement debate. Moreover, whenever specific biomedical interventions are discussed, it is particularly worrisome that surprisingly little attention is given to side-effects, risks and safety-issues. Every biomedical intervention in the brain will likely have unintended, unwanted or unexpected side effects, especially so in cases where the underlying mechanisms of action are not well-understood and/or the procedure is invasive.

While neurofeedback and transcranial stimulation are non-invasive procedures, electrical stimulation of the brain via electrode implants and neuron replacement therapy are highly invasive. For example, aside from risks associated with brain surgery and electrode placement (e.g. brain hemorrhage, infection, death), DBS for Parkinson’s disease carries a 1.1-33% risk of cognitive side effects (e.g. speech disturbance), a 1.3-10.2% risk of behavioral side effects (e.g. hypomania), a 0.5-25% risk of psychiatric side effects (e.g. depression) and a 50-71% risk of familial side effects (e.g. marital problems) [93]. While electrical stimulation of the brain via electrode implants is essentially reversible, neuron replacement therapy is a non-reversible procedure involving the injection of stem cells into the brain or spinal cord. Aside from the risks associated with surgery and stem cell injections, this carries the risk of tumor growth, seizures or intractable pain [94]. Finally, even more familiar interventions such as pharmaceuticals and hormones have risks and side-effects, and their long term effects are not always known. In sum, in contrast to what has been the case so far, safety should be a key issue in the debate on moral enhancement.

### Identity changes

It is particularly surprising from a philosophical point of view that so little attention is given in the debate to unintended, unwanted or unexpected identity changes and the huge impact these changes may have on one’s self-understanding, well-being and social and familial relationships. This problem has been discussed quite extensively in relation to psychopharmaceuticals (especially SSRI’s) and DBS, but potential identity changes due to changes in one’s moral dispositions or behavior are not often touched upon in the moral enhancement debate.

Within the broader biomedical treatment and enhancement debate, several authors have convincingly argued that whereas drastic identity changes are problematic from an ethical perspective, typically requiring the discontinuation of the treatment or intervention, mild or moderate changes are not necessarily ethically problematic (e.g. [95]). The key philosophical concept that is at stake is the concept of narrative identity rather than numerical identity. Narrative identity reflects an individual’s most central and salient characteristics (e.g., motivations, beliefs, values, desires, character traits) that together comprise their self, and needs to be understood within the dynamics of psychological change. These characteristics may and often do change throughout one’s life in response to various life events. It is important for the continuity of narrative identity that such changes are or can be incorporated into one’s life story in a coherent way, without compromising one’s sense of self. Since many if not most of such ‘naturally’ or ‘traditionally’ occurring changes are experienced in a non-problematic and identity preserving way throughout our life, one can, by comparison, argue for the ethical acceptability of narrative identity changes resulting from biomedical treatment or enhancement interventions [96,97]. At the same time however, it could be the case that unforeseen, instantaneous, uncontrollable and/or drastic changes in one’s *moral* dispositions or behavior are more likely to disrupt one’s narrative identity, giving rise to a whole new array of personal and even societal queries. In agreement with the latter view, in a public opinion study on biomedical enhancement, individuals reported to be most reluctant to undergo enhancements of traits that are more fundamental to the self (e.g., morally relevant traits such as empathy and kindness) and the most frequently voiced reasons for resisting those enhancements were concerns of changes to their fundamental self [98].

## Summary

In this paper, we have categorized and discussed the arguments in the published debate on the ethical desirability of moral bioenhancement. We have organized these arguments under the following headings: (1) why we (don’t) need moral bioenhancement, (2) it will (not) be possible to reach consensus on what moral bioenhancement should purport, (3) the feasibility of moral bioenhancement and the status of current scientific research, (4) means and processes of arriving at moral improvement matter ethically, (5) arguments related to the freedom, identity and autonomy of the individual, and (6) arguments related to social/ group effects and dynamics.

After discussing each argument in more detail, we have identified a number of issues that in our view merit greater attention. First, we observed that, in the debate so far, discussions about the moral enhancement of ‘humanity as a whole’ and the targeted treatment of specific mental health disorders (such as psychopathy) are not sufficiently distinguished. Many authors overestimate the scientific as well as the practical feasibility of the interventions they discuss, rendering the debate too speculative. Related to this is our observation that insufficient attention is devoted to possible side-effects, risks and safety. There is also remarkably little attention to questions about identity and identity change.

We believe that the debate on moral enhancement is extremely interesting from a meta-ethical point of view, since it triggers important questions about the nature of morality, moral thought and moral behavior. However, the normative ethical question as to whether moral bioenhancement *as such* is good or bad, desirable or not, is not a very fruitful question for further debate. We therefore believe that, instead of speculating about non-realistic scenarios like the genetic engineering of morality, or other imaginary forms of biomedical moral enhancement of ‘the whole of humanity’, it would be much more useful to discuss novel and emerging biomedical interventions that may improve moral capacities or moral behavior in specific target groups and in relation to particular mental health problems. For the near future, biomedical treatment of moral pathologies may be a more realistic option than moral enhancement, and may raise more concrete moral questions.

In order for the moral enhancement debate to move beyond its focus on speculative philosophical theorizing and discussion, we need in-depth analyses of both the practical feasibility of existing or novel biomedical interventions for moral therapy (and perhaps eventually enhancement); and the ethical acceptability of such interventions, including safety concerns.

We conclude that although the discussion on moral enhancement so far raises interesting questions on an abstract, philosophical level, it often appears to be too remote from real (and realistic) contexts and applications to do justice to the specific ethical questions raised by such practices. We therefore urge for a more focused debate on realistic options of biomedical treatment of moral pathologies and the concrete moral questions these treatments raise.

## Competing interests

The authors declare that they have no competing interests.

## Authors' contributions

All authors were involved in the conception and design of the study. JS was involved in the data gathering and analysis, in drafting the manuscript and in the writing of the paper. FF was involved in writing of the paper. KR was involved in the data gathering and analysis. MS was involved in the data analysis and writing of the paper. All authors commented on sequential drafts. All authors read and approved the final manuscript.

## Pre-publication history

The pre-publication history for this paper can be accessed here:

http://www.biomedcentral.com/1472-6939/15/67/prepub

## Supplementary Material

Additional file 1 Flow chart. Click here for file
