# Supplementary figures and images for: The ethical desirability of moral bioenhancement: a review of reasons
Source: BMC Med Ethics. 2014 Sep 16;15:67. doi: 10.1186/1472-6939-15-67 (PMC4274726; doi:10.1186/1472-6939-15-67)

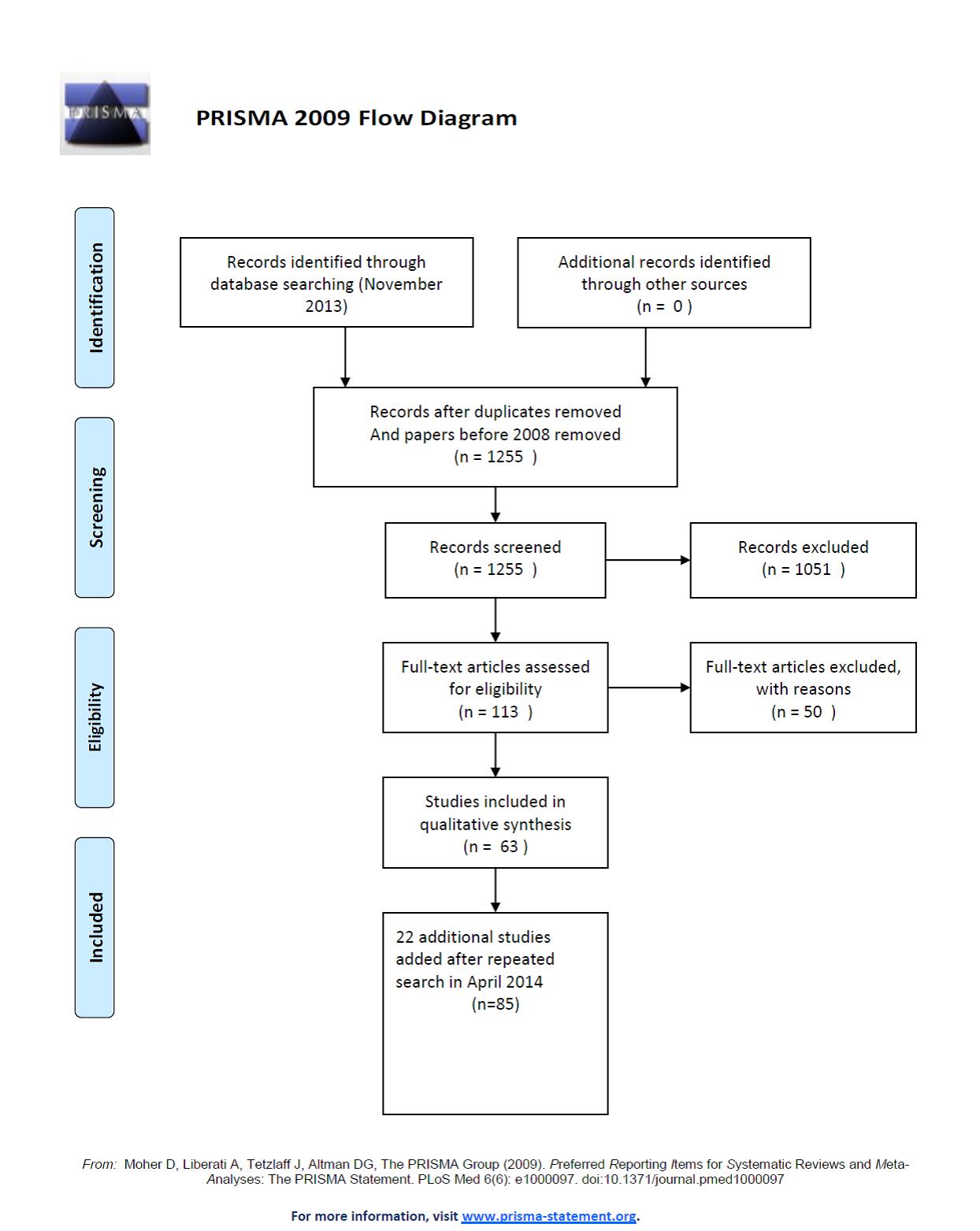

Supplement: Additional file 1 — Flow chart. [file 1472-6939-15-67-S1.jpg]
